# Comparison of open ovariectomy with sutures or bipolar vessel sealing versus laparoscopic approach in cats

**DOI:** 10.1007/s11259-025-11039-6

**Published:** 2026-01-12

**Authors:** Marta Guadalupi, Claudia Piemontese, Marzia Stabile, Caterina Vicenti, Alberto Maria Crovace, Francesco Staffieri, Luca Lacitignola

**Affiliations:** 1 Sez. Cliniche veterinarie e p.a., Dipartimento DiMePRe-J, Campus di Medicina Veterinaria, Università Degli Studi di Bari “Aldo Moro”, s.p. per Casamassima km 3, Valenzano, Bari, 70010 Italy; 2 Dipartimento di Medicina Veterinaria, Università degli Studi di Sassari, Via Vienna, 2, Sassari, 07100 Italy

**Keywords:** Cat, Ovariectomy, Laparoscopy, Bipolar vessel sealing device, Post-operative pain

## Abstract

To compare surgical time, incision length, intraoperative bleeding, and post-operative pain in cats undergoing three different ovariectomy techniques: open surgery with pedicle ligation using sutures, open surgery using a bipolar vessel-sealing device, and a two-port laparoscopic approach. A prospective randomized clinical trial was conducted on 27 healthy female cats assigned to three treatment groups (*n* = 9 per group). Surgical variables were recorded intraoperatively, and post-operative pain was assessed using a validated feline pain scale at hourly intervals over four hours. The laparoscopic group had the shortest incision length (mean 10.0 mm, SD 0.0) compared to the suture (mean 33.3 mm, SD 5.6) and bipolar device groups (mean 28.7 mm, SD 6.4). Surgical time was significantly shorter in the BVSD (27.0 ± 9.6 min) and LOVE groups (30.2 ± 5.2 min) compared with the Suture group (43.9 ± 14.4 min; one-way ANOVA, *p* = 0.005; Tukey post-hoc *p* < 0.05 vs. Suture for both comparisons). Post-operative pain scores at one hour (T1) were lower in the LOVE group (median 4 [IQR 3–5]) than in both open groups (Suture: 9 [IQR 8–9]; BVSD: 7 [IQR 6–8]; Kruskal–Wallis, *p* = 0.014; Dunn’s post-hoc *p* < 0.05 vs. BVSD and trend towards lower scores vs. Suture). Only 1 of 9 cats requiring rescue analgesia versus 7 of 9 in each open group. The laparoscopic approach was associated with lower post-operative pain scores and a reduced need for rescue analgesia compared to open ovariectomy techniques, suggesting improved perioperative comfort. Although the laparoscopic group showed a significantly shorter surgical time compared with the suture group, this observation should be interpreted cautiously due to potential operator- and case-dependent variability. Nevertheless, laparoscopic ovariectomy in cats should be considered a promising and welfare-oriented technique that warrants further investigation.

## Introduction

Ovariectomy is a surgical procedure that is routinely performed for the elective sterilization of female cats. While conventional open approaches remain widely practiced, minimally invasive surgery (MIS) is increasingly widespread in veterinary surgery due to the potential advantages in terms of improved intraoperative visualization of anatomical structures (GAUTHIER et al. 2015; Hancock et al. 2005; Mayhem and Brown 2007; Sakals et al. 2018), lower tissue trauma (Hancock et al. 2005; Sakals et al. 2018), and lower risk of surgical site infections (Sakals et al. 2018). Despite this, MIS has some disadvantages that include the technical expertise required, the learning curve involved, and the cost of specialized equipment.(Hancock et al. 2005; Sakals et al. 2018).

It is important to consider that the laparoscopic technique requires a longer learning curve than open techniques, however, in dogs a high variability in the learning curve for laparoscopy has been reported (8 to 80 procedures). (Pope and Knowles 2014; Runge et al. 2014) Data specific to feline patients remain scarce. In a recent small case series on laparoscopic ovariectomy in cats, novice operators demonstrated progressive improvements in operative time and procedural fluency over the course of only a few procedures. (BEL et al. 2023) This suggests a potentially shorter and more manageable learning curve in cats, likely due to differences in size, tissue handling, and anatomical exposure.

From an economic perspective, it should be noted that laparoscopic ovariectomy requires specific equipment and instruments (such as a laparoscopic tower, trocars, and vessel-sealing devices) which represent an initial investment for the facility; however, these costs tend to be amortized over time with increasing surgical caseloads. Although the procedure may involve a slightly higher cost for the owner, the potential advantages in terms of reduced postoperative pain, faster recovery, and improved patient comfort may justify this difference, supporting the gradual adoption of laparoscopy in feline clinical practice.

Several studies that compared the laparoscopic ovariectomy (LOVE) with open surgical techniques in female dogs and cats are reported in current literature (Case et al. 2015; GAUTHIER et al. 2015; Hancock et al. 2005; Sakals et al. 2018). These studies suggest that laparoscopic techniques were associated with less post-operative pain, compared to open techniques.

During ovariectomy, one critical step influencing both surgical duration and nociceptive response is the method used to achieve haemostasis of the ovarian pedicle.(Pereira et al. 2018) In traditional open surgery, this is typically achieved through suture ligation. (Case et al. 2015; Gauther et al. 2015; MAYHEW and BROWN 2007) However, a variety of hemostatic modalities have been employed for laparoscopic gonadectomy, including the use of surgical steel clips, harmonic scalpel, laser, and monopolar or bipolar electrosurgical devices, and extracorporeal sutures. (Austin et al. 2003; DAVIDSON et al. 2004; Devitt et al. 2005, Gauther et al. 2015; Hancock et al. 2005; MAYHEW and BROWN 2007; Van Goethem et al. 2003, VAN NIMWEGEN and KIRPENSTEIJN 2007; Watts 2018). Among these, bipolar vessel-sealing devices (BVSDs) have demonstrated efficacy and safety in achieving pedicle hemostasis in various species, including horses, dogs, and cats, while also contributing to decreased operative times and improved intraoperative control; however, only a few studies (HENIFORD et al. 2001; Sakals et al. 2018; VAN NIMWEGEN and KIRPENSTEIJN 2007; Coisman et al. 2014) have specifically addressed this topic, and systematic comparisons in feline laparoscopic or open ovariectomy remain scarce.

To our knowledge, few studies have systematically compared these methods in feline laparoscopic or open ovariectomy, and data specific to cats remain sparse.

The aim of the current study was to compare surgical time, incision length, intraoperative bleeding, and post-operative pain associated with the three different ovariectomy surgical procedures in cats: open OVE with standard ovarian pedicle ligation sutures, open OVE using the BVSD, and LOVE.

Our hypothesis was that the laparoscopic approach for ovariectomy in cats reduces surgical duration, incision length, intraoperative bleeding, and post-operative pain compared with open techniques.

## Materials and methods

### Study design

Prospective randomized clinical study.

### Animal welfare and ethical committee

The current study was approved by the Ethical Committee for Clinical and Zootechnical Studies of the Department of Precision and Regenerative medicine and the Ionian area (DiMePre-J), University of Bari “Aldo Moro” (protocol n° 03/2021). The owners of all the animals included in the present study were provided with information regarding the study and written informed consent was obtained.

### Animal and experimental groups

For the purpose of this study female cats were enrolled prospectively from April 2024 to April 2025. The selected cats were randomly divided into three groups based on the method employed to perform the ovariectomy procedure. For two groups, a standard midline approach to the abdominal cavity was performed: Suture group, in which the ovarian pedicle was ligated by means of a suture, and Bipolar Vessel Sealing Device (BVSD) group, in which the ovarian pedicle was resected using a bipolar vessel sealing device; whereas a two-port laparoscopic approach was used for the third group (LOVE group). Randomization was performed using an online program (www.random.org).

### Inclusion and exclusion criteria

Subjects were considered suitable for the study on the basis of the following inclusion criteria: age ranging from six months to five years, healthy, no signs of oestrus, no litter in the past 60 days, and not pregnant. All selected patients underwent clinical examination and basic haematological testing (complete blood count, alanine aminotransferase, serum creatinine, and total protein), and only cats with no clinically relevant abnormalities were deemed eligible and enrolled in the study. Subjects who did not meet the inclusion criteria were excluded.

### Anaesthetic protocol

All the subjects included in the present study were premedicated by an intramuscular injection of dexmedetomidine (5 µg/kg, Dexdomitor, Vetoquinol, Bertinoro, Italy), alfaxalone (1 mg/kg; Alfaxan, Dechra Veterinary Products Srl, Turin Italy), and buprenorphine (10 µg/kg, Buprenodale, Dechra Veterinary Products Srl, Turin Italy) combination, after a minimum six hours of fasting. Following a level of sedation sufficient to allow safe handling, an intravenous access was placed through one of the cephalic veins, in order to administer fluids and drugs. A subcutaneous (SC) dose of robenacoxib (2 mg/kg) and amoxicillin/clavulanic acid (20 mg/kg; SC) were administered to all the cats. General anesthesia was induced using alfaxalone, administered to effect until both the palpebral reflex and jaw tone were lost, and maintained by means of the inhalation of isoflurane (Isoflurane Vet, Boehringer Ingelheim Animal Health Italia S.p.A., Padova, Italy) carried by pure oxygen. Following the desensitization of the laryngeal area by means of one or two puffs of a 1% solution of lidocaine (Lidocaina Cloridrato Salf 1%, Salf S.p.A. Laboratorio Farmacologico, Bergamo, Italy), all the subjects were intubated using an orotracheal tube of internal diameter 3–3.5 mm. The cats were connected to a non-rebreathing circuit and received a fresh gas flow rate of 300 ml/kg during the entire duration of the surgical procedure. Spontaneous breathing was maintained throughout the procedure. Following the attainment of an adequate level of anaesthesia, all the cats received a constant rate of infusion of 1 µg/kg/h of dexmedetomidine, until the completion of the procedure. Uniform protocols are applied to ensure methodological consistency and reproducibility across all experimental groups. The dosage of isoflurane was adjusted in accordance with the depth of anaesthesia, which was assessed on the basis of the observation of eyelid reflex, eye position, and the tone of the jaw muscles, along with the respiratory rate (RR, breaths/minute), heart rate (HR, beats/minute), and mean arterial pressure (MAP, mmHg). During anesthesia, the following physiological parameters were continuously monitored (S5 multiparametric anaesthesia monitor; Datex Ohmeda): HR, MAP, RR, peripheral oxygen saturation (SpO_2_, %), partial pressure of end-tidal carbon dioxide (EtCO_2_, mmHg), and rectal temperature (T, °C). During the entire surgical procedure, the cats received an intravenous infusion of ringer lactate solution at the rate of 3 mL/kg/h. HR, MAP and RR were continuously monitored and compared with individual baseline values recorded after induction and stabilization. A sustained increase of **≥ 20%** above baseline in any of these parameters, persisting for > 1 min and not attributable to inadequate anesthetic depth, was interpreted as intraoperative nociception and treated with **fentanyl 2 µg/kg IV** as rescue analgesia. This criterion is consistent with published feline studies on ovariohysterectomy and intraoperative monitoring (BELLINI et al. 2017; Interlandi et al. 2024; Lima et al. 2024; Moretti et al. 2024) and with guideline statements on recognizing autonomic responses as indicators of nociception under general anesthesia (Robertson et al. 2018).

After the completion of the surgical procedure, the administration of dexmedetomidine and isoflurane was discontinued, and the cats were allowed to recover with adequate heating and fluid support. All cats were discharged 4 h after extubation, immediately after completion of all postoperative assessments.

### Surgical procedure in the suture group

Open procedures were performed by a surgeon in training (PhD candidate with clinical rotation in small animal surgery) under the supervision of a senior surgeon. All critical steps, such as vascular pedicle ligation and hemostasis verification, were supervised or assisted by the senior surgeon to ensure procedural consistency and safety.

The ventral abdominal area was aseptically prepared, and the patients were positioned in dorsal recumbency. Then, a median incision was made through the skin and linea alba. Subsequently, the left uterine horn was exteriorized manually. The ovarian pedicle was ligated using 3 − 0 USP polyglactin 910 (Vicryl, Ethicon, Cincinnati, OH). Standard technique was applied, with ligatures placed on the pedicle adjacent to the ovary and the ovary subsequently excised with Metzenbaum scissors. The same procedure was performed on the contralateral ovary. Muscles and fascia were sutured in a single continuous pattern using 0 USP polyglactin 910 (Vicryl, Ethicon, Cincinnati, OH). The skin was closed using intradermal sutures (3 − 0 USP Vicryl rapid, Ethicon, Cincinnati, OH).

### Surgical procedure in the BVSD group

The same surgical team of the previous group performed the surgical procedure similarly to the aforementioned procedure in the Suture group, with the exception of the ovarian pedicle ligation. The vessel-sealing device for open surgery (LigaSure Small Jaw, Covidien, Milan, Italy) was used to coagulate and divide the ovarian pedicle and uterine horn at the junction with the proper ovarian ligament. The power of the instrument was adjusted to level 2 for all cases.

### Surgical procedure in the LOVE group

Laparoscopic procedures were performed by a senior surgeon (Professor of Veterinary Surgery with > 15 years of clinical and teaching experience in minimally invasive surgery), with direct assistance from the same surgeon in training involved in the open procedures. To minimize variability related to operator experience, only the critical phases of trocar insertion and initial abdominal access were exclusively performed by the senior surgeon, while the remaining steps followed a standardized protocol identical across all subjects.

Patients were initially prepared aseptically after adequate trichotomy of the ventral area of the abdomen and then positioned in dorsal decubitus.

According to the modified Hasson technique, the procedure required a 5 mm incision along the midline of the abdomen about 1 cm caudal to the umbilicus. Once the trocar was successfully installed, the abdomen was insufflated with CO_2_ at maximum pressure of 4 mmHg. Then the telescope (5 mm, 0°, 29 cm Hopkins II laparo- scope, Karl Storz Endoscope) was introduced through this port.

After abdominal exploration was performed, the second instrument port was created midway between the xiphoid cartilage and the umbilicus, using a 5 mm threaded cannula.

The cat is then rotated 45° to achieve a left lateral decubitus to allow easier identification of the right ovary. The right ovary is grasped by a laparoscopic Babcock forceps and then lifted to be able to optimize visualization of the ovarian vasculature, the adnexal ligaments, and specifically the ovary proper ligament and the suspensory ligament.

Therefore, under laparoscopic guidance, a transabdominal suspension suture was placed with nylon USP 0 with 36 mm long half circle needle and positioned in order to suspended ovary and isolate the ovarian pedicle.

A 5-mm endoscopic bipolar vessel-sealing device (LigasureTM, Covidien, Mansfield, MA or Caiman BBRAUN; Milan, Italy) is inserted through the instrumental port. Once this procedure was completed, the ovary was extracted through the operative trocar. The contralateral ovariectomy was performed after placing the cat in the opposite 45° lateral recumbency and performed similarly to the opposite ovary.

The cat is then rotated in dorsal recumbency, the pneumoperitoneum was suspended and CO2 evacuated from the abdomen. The portal sites were closed with two layers: the abdominal musculature with noncontinuous absorbable multifilament suture (Polyglactin 910 USP 0), and the skin with absorbable interrupted horizontal mattress pattern sutures (Polyglactin 910 USP 2 − 0).

The vessel-sealing device employed for BVSD and LOVE groups were reprocessed with ethylene oxide according to institutional sterilization standards and were used uniformly across all surgical groups.

### Surgical variables and complications

In the present study, the length of the surgical incision in both the groups was measured after skin closure using a millimetric caliber.

The duration of the surgical procedure in the Suture, BVSD and LOVE groups was defined as the time interval between the commencement of the first skin incision and the placement of the last suture (skin-to-skin).

Bleeding from the ovarian bursa, ovarian pedicle, or the proper ligament was scored as follows: 0: no bleeding; 1: mild bleeding with a few drops of blood that stopped immediately; 2: moderate bleeding that did not obscure the surgical field but required cauterization; 3: severe bleeding that impeded adequate visualization of the surgical field and required clamping and cauterization.

### Pain assessment

Post-operative pain was assessed during the immediate recovery period, at 1, 2, 3, and 4 h after extubation, using the Glasgow Feline Composite Measure Pain Scale (CMPS-Feline) (Reid et al. 2017). Post-operative pain was assessed during the immediate recovery period at 1, 2, 3 and 4 h after extubation using the CMPS-Feline. Prior to the first pain assessment, at approximately 30 min after extubation, a standardised sedation/recovery check was performed by the same observer to ensure adequate arousal and mobility (e.g., ability to lift head, respond to verbal stimuli, maintain sternal posture) before application of the CMPS-Feline. Only cats meeting this criterion proceeded to the pain scoring sequence. Assessments were performed by a single observer blinded to the surgical technique, and rescue analgesia (tramadol 3 mg/kg IM) was administered whenever the CMPS-Feline total score reached or exceeded the established intervention threshold of 5 out of 20 points. This time frame was selected because all procedures were performed on an outpatient basis, and cats were discharged from the hospital once full recovery from anesthesia was achieved. Consequently, pain assessment beyond 4 h could not be performed under standardized conditions.

### Post-operative regimen and after care

Once fully awake, normothermic, and able to ambulate, patients were discharged on the same day, in accordance with the outpatient nature of the procedures. Owners were instructed to inspect the surgical wound daily and to monitor for appetite, behavior, and normal urination/defecation. The ingestion of food and water was permitted after a period of eight hours following the recovery from anesthesia. A clinical re-evaluation was scheduled at 7 days post-operatively, coinciding with suture removal, and again at 14 days to confirm uneventful healing. Post-operative antimicrobial and analgesic medications were administered using amoxicillin/clavulanic acid (20 mg/kg per OS, BID, for three days; Synulox, Zoetis Italia) and robenacoxib (1 mg/kg per OS, SID, for three days; Onsior, Novartis). The surgical wound was cleaned and disinfected by the owner on a daily basis using a solution of 0.05% chlorhexidine solution for a time period of one week after the surgery.

### Statistical analysis

Statistical analyses were performed using jamovi (The jamovi project, 2023). Data were summarized as mean ± standard deviation (SD) for normally distributed continuous variables or as median [IQR] for non-normally distributed or ordinal data. Normality was assessed using the Shapiro–Wilk test prior to selecting the appropriate analysis.

A one-way analysis of variance (ANOVA) was applied to evaluate differences in age, body weight, incision length, and surgical duration among the Suture, BVSD, and LOVE groups, as these variables were continuous and normally distributed. Intraoperative complications were analyzed qualitatively and did not include any continuous variable suitable for ANOVA. Hemorrhage scores, recorded on an ordinal scale (0–3), were analyzed using the Kruskal–Wallis test followed by Dunn’s post-hoc test when appropriate. Postoperative pain scores (Glasgow CMPS-F) were ordinal and non-normally distributed; therefore, comparisons between groups at each time point (T1–T4) were performed using the Kruskal–Wallis test with Dunn’s post-hoc correction, while within-group changes over time were analyzed using the Wilcoxon signed-rank test. Results are presented as median [IQR].

Hemorrhage scores, recorded on a 2-point ordinal Likert scale (0 = no bleeding, 1 = mild bleeding), were treated as non-parametric ordinal data and analyzed using the Kruskal–Wallis test followed by Dunn’s post-hoc test when appropriate. These variables were not considered continuous, and therefore ANOVA was not applied to this dataset.

A priori power analysis was based on preliminary data from an internal pilot study conducted to estimate the effect size for surgical duration, chosen as the primary quantitative endpoint for sample size determination. The analysis assumed a minimum clinically relevant difference of approximately 15 min in surgical time among groups, with an expected standard deviation of about 10 min (corresponding to a Cohen’s f of approximately 0.40) in a one-way ANOVA design. The analysis was performed with a statistical power of 80% (β = 0.20) and a significance level of 0.05 (α = 0.05), indicating that a minimum of nine animals per group would be sufficient to detect significant differences among treatment groups. Consequently, a total of 27 subjects (9 per group) was established as the sample size for the present randomized clinical trial.

## Results

### Animals

The present study involved 27 healthy, intact, female cats of the European breed (*n* = 9/group).

The mean ages with 95% confidence intervals (CI) of the subjects in the Suture, BVSD, and LOVE groups were 11.22 months (CI: 8.47–13.97), 17.00 months (CI: 8.51–25.49), and 14.78 months (CI: 8.36–21.19), respectively.

The mean body weights with 95% CI for the Suture, BVSD, and LOVE groups were 3.14 kg (CI: 2.86–3.43), 3.16 kg (CI: 2.77–3.54), and 3.20 kg (CI: 2.94–3.46), respectively (Table 1).

**Table 1:** Descriptive statistics for age and body weight across the Suture, BVSD and LOVE groups: open ovariectomy with suture ligation (Suture), open ovariectomy with bipolar vessel-sealing device (BVSD), and laparoscopic ovariectomy (LOVE). Data are presented as mean, 95% confidence interval (lower and upper bounds), median, and standard deviation (SD)

			95% Confidence Interval			
	group	Mean	Lower	Upper	Median	SD
AGE (months)	BVSD	17.00	8.51	25.49	12.00	13.000
	SUTURE	11.22	8.47	13.97	9.00	4.206
	LOVE	14.78	8.36	21.19	12.00	9.821
WEIGHT (kg)	BVSD	3.16	2.77	3.54	3.00	0.588
	SUTURE	3.14	2.86	3.43	3.00	0.439
	LOVE	3.20	2.94	3.46	3.20	0.391

**Fig. 1:**
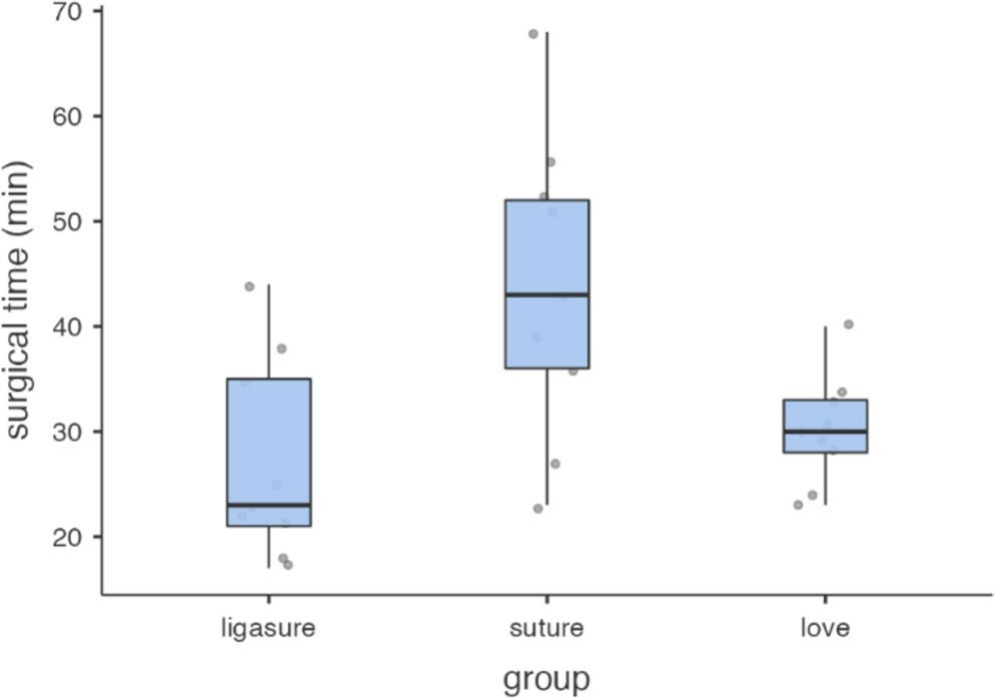
Plot depicting the duration of surgery pertaining to the Suture, the BVSD and the LOVE groups. A statistically significant difference (*p* < 0.05) was observed between the Suture, the BVSD and the LOVE groups; while no statistically significant difference was found between the LOVE group and the BVSD group

No significant differences were observed in age (F(2, 13.1) = 1.11, *p* = 0.359) or body weight (F(2, 15.6) = 0.04, *p* = 0.958) among the Suture, BVSD and LOVE experimental groups. These findings indicate that the subjects were homogeneous in terms of baseline characteristics, confirming an adequate randomization and comparability across treatment groups (Fig. 1).

### Surgical variables

All continuous variables (incision length and surgical time) were normally distributed, as confirmed by the Shapiro–Wilk test (all *p* > 0.05). Therefore, data are presented as mean ± SD, and group comparisons were performed using one-way ANOVA followed by Tukey’s post-hoc test.

For incision length, significant differences were found among the Suture, BVSD, and LOVE groups (*p* < 0.001). The LOVE group showed the shortest incision length (10.0 ± 0.0 mm), consistent with the standardized two-port laparoscopic approach (5 mm + 5 mm). The BVSD group exhibited intermediate values (28.7 ± 6.4 mm), while the Suture group had the longest incisions (33.3 ± 5.6 mm). Post-hoc analysis confirmed that incision length was significantly shorter in the LOVE group compared to both the BVSD (*p* < 0.001) and Suture (*p* < 0.001) groups, whereas the difference between BVSD and Suture was not significant (*p* = 0.129).

Regarding surgical time, the mean ± SD values were 43.9 ± 14.4 min for the Suture group, 27.0 ± 9.6 min for the BVSD group, and 30.2 ± 5.2 min for the LOVE group. One-way ANOVA revealed a statistically significant difference among groups (*p* = 0.005). Post-hoc Tukey comparisons showed that surgical time was significantly longer in the Suture group compared to both BVSD (mean difference − 16.9 min, *p* = 0.006) and LOVE (mean difference − 13.7 min, *p* = 0.027), while no significant difference was detected between the BVSD and LOVE groups (mean difference − 3.2 min, *p* = 0.791). (Table 2; Fig. 1).

**Table 2:** Comparison of surgical time (minutes) among groups.Values are expressed as mean ± SD. Differences were analyzed by one-way ANOVA followed by tukey’s post-hoc test

Group	*n*	Surgical time (Min, Mean ± Sd)	
Suture	9	43.9 ± 14.4	Suture > BVSD (*p* = 0.004); Suture > LOVE (*p* = 0.02)
BVSD	9	27.0 ± 9.6	ns vs. LOVE (*p* > 0.5)
LOVE	9	30.2 ± 5.2	

Intraoperative bleeding was minimal in all cases and easily controlled without complications.

In the Suture group, mild bleeding was observed in 8 subjects and absent in 1 subject. In the BVSD group, 3 subjects showed mild bleeding while 6 had no bleeding. In the LOVE group, no bleeding was recorded in any subject.

**Fig. 2:**
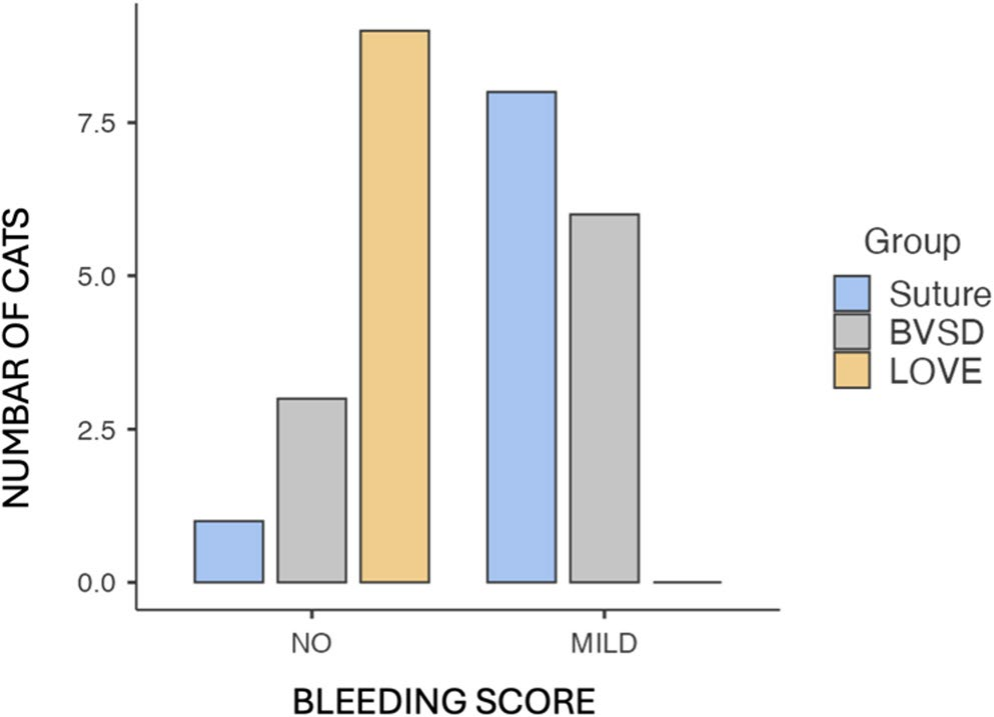
Plots of the bleeding degree for the Suture, BVSD and LOVE groups. Bleeding was classified as “NO” (score = 0) or “MILD” (score = 1)

Bleeding scores, classified as no (score = 0) or mild (score = 1), differed significantly among groups (Kruskal–Wallis, *p* < 0.001). Post-hoc analysis revealed that the LOVE group presented significantly lower bleeding scores compared with both the Suture and BVSD groups (both *p* < 0.001), while no significant difference was detected between Suture and BVSD. These findings indicate superior intraoperative hemostasis with the laparoscopic (LOVE) technique. (Fig. 2).

### Pain

Intraoperative rescue analgesia was not required in any animal across the Suture, BVSD, and LOVE groups. All cats met the predefined recovery status checkpoint prior to the first pain evaluation, ensuring consistency of the timing and conditions under which CMPS-Feline scoring was applied. Postoperative pain was evaluated using the CMPS-F at 1 (T1), 2 (T2), 3 (T3), and 4 (T4) hours after extubation.

The distribution of pain scores was tested for normality using the Shapiro–Wilk test, which indicated non-normal data in several subgroups (e.g., Suture T1, Ligasure T4). Consequently, pain data were analyzed using nonparametric tests and are reported as median [IQR].

At T1, before the administration of any rescue analgesia, pain scores differed significantly among groups (Kruskal–Wallis, *p* = 0.014). The LOVE group showed significantly lower values than the BVSD group (Dunn’s post-hoc, *p* < 0.05), while the difference between LOVE and Suture approached but did not reach statistical significance (*p* = 0.065).

No significant differences were detected among groups at T2, T3, or T4 (all *p* > 0.05). The lack of intergroup differences at later time points likely reflects the confounding effect of rescue analgesia, which was administered to most cats in the Suture and BVSD groups immediately after T1.

**Fig. 3:**
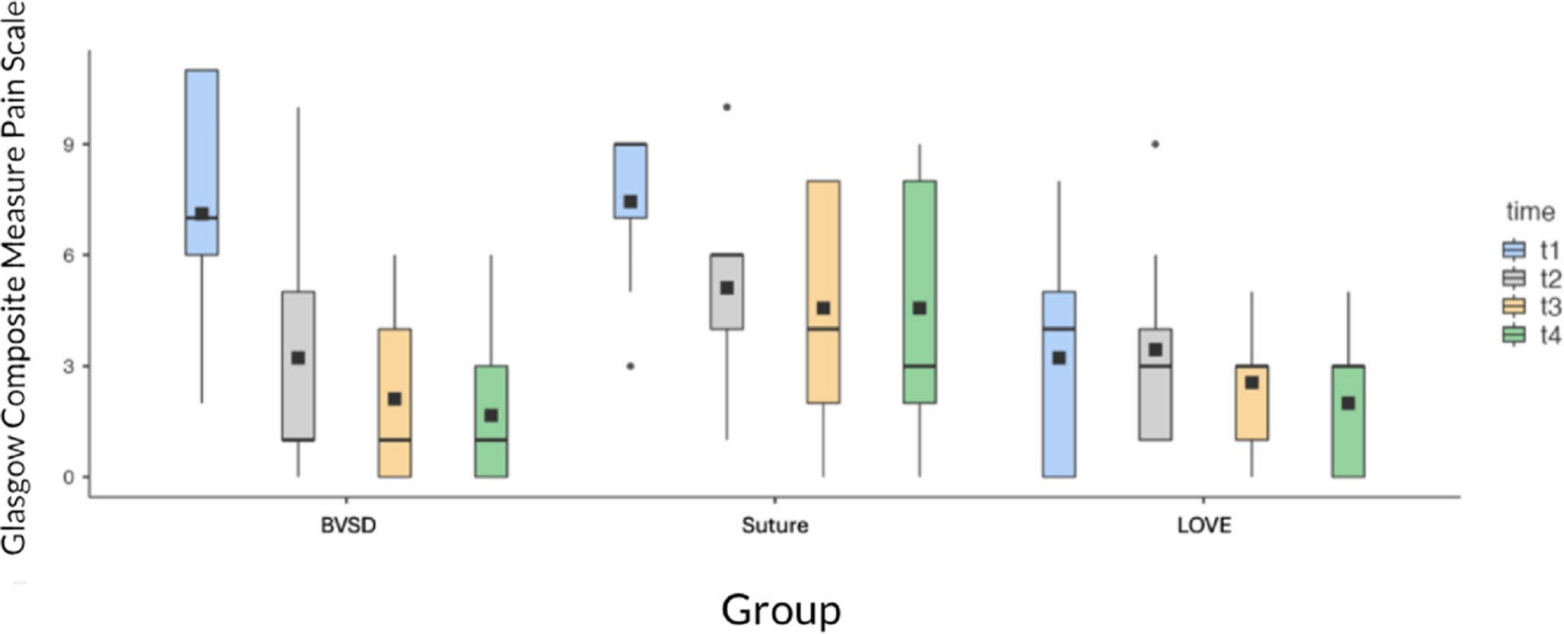
Box-plot of post-operative pain scores assessed using the Glasgow Composite Measure Pain Scale for Felines in the BVSD, Suture, and LOVE groups at four time points (T1–T4), corresponding to 1, 2, 3, and 4 h after extubation, respectively. At T1, prior to the administration of rescue analgesia, pain scores were significantly higher in both the Suture and BVSD groups compared with the LOVE group (Dunn’s post-hoc, *p* < 0.05). Pain scores decreased over time in all groups, but remained consistently lower in the LOVE group throughout the observation period

Within-group analysis (Wilcoxon signed-rank test) confirmed a significant reduction in pain scores from T1 to T4 in both Suture (*p* = 0.049) and BVSD (*p* = 0.004), while the LOVE group showed no significant variation (*p* > 0.2). (Fig. 3).

## Discussion

The findings of the present study support our hypothesis that the laparoscopic approach in cats undergoing ovariectomy is associated with a significant reduction in post-operative pain scores and intraoperative bleeding, as well as a shorter surgical time and incision length compared to the traditional suture ligation technique, although no significant difference in surgical duration was observed when compared with the BVSD group.

### Surgical variables

Regarding surgical variables, operative times were found to be significantly shorter in both the BVSD and LOVE groups compared to the Suture group, whereas no statistically significant difference was observed between the BVSD and LOVE groups. In our opinion, these results highlight that the duration of ovariectomy procedures is significantly reduced when a vessel-sealing device is employed. Furthermore, in our study, performing the procedure via the laparoscopic approach did not contribute to a longer surgical time compared to traditional laparotomy.

The use of vessel-sealing devices in laparoscopic OVE and OVH has been described in dogs (Öhlund et al. 2011) and cats (VAN NIMWEGEN and KIRPENSTEIJN 2007). The reduction in the duration of surgery with regard to the ovarian pedicle dissection is one of the main features that facilitated the widespread diffusion of this surgical technique among veterinary surgeons (KATIĆ and DUPRÉ, 2017; SCHWARZKOPF et al. 2015). In particular, a previous study by KIM et al. (2011) demonstrated the feasibility of single-port laparoscopic ovariectomy in cats, supporting the concept that minimally invasive approaches can be performed safely in this species and reinforcing the applicability of our findings to different laparoscopic settings. (KIM et al. 2011)

The use of a BVSD allowed efficient transection of the ovarian pedicle and provided consistent hemostasis without prolonging surgical time. The LigaSure™ system combines bipolar energy with controlled mechanical compression to produce secure vessel sealing for vessels up to 7 mm in diameter (KARANDE 2015).

In the current study, suture ligation of the ovarian pedicle was associated with a higher frequency of mild intraoperative bleeding compared to BVSD and LOVE. This difference was statistically significant (Kruskal–Wallis, *p* < 0.001), with the LOVE group showing significantly lower bleeding scores than both the Suture and BVSD groups, consistent with the results reported.

Although these episodes were clinically insignificant and easily controlled, they temporarily impaired visualization and required additional maneuvers, thereby contributing to slightly longer operative times observed in the open surgery groups. (MAYHEW and BROWN 2007).

Maintaining a low insufflation pressure during laparoscopy was a deliberate methodological choice aimed at preserving spontaneous ventilation and minimizing any alteration in ventilatory or hemodynamic homeostasis. In the present study, the pneumoperitoneum was established and maintained at a maximum intra-abdominal pressure (IAP) of 4 mmHg. This setting was selected to ensure an adequate surgical field while preventing potential respiratory compromise, which may occur in cats due to their relatively small abdominal volume and highly compliant abdominal wall. Previous experimental and clinical studies have demonstrated that higher insufflation pressures (> 8 mmHg) offer minimal advantages in terms of workspace expansion, while increasing the risk of altered pulmonary mechanics and reduced venous return (Mayhew et al. 2013). Conversely, laparoscopic ovariectomy in feline patients has been successfully performed at 4 mmHg without the need for assisted ventilation, maintaining normocapnia and stable hemodynamic parameters throughout the procedure(VAN NIMWEGEN and KIRPENSTEIJN 2007). A pressure–volume analysis further shows diminishing returns in abdominal volume beyond ~ 8 mmHg and highlights the importance of avoiding unnecessarily high IAPs in small animals (Dorn et al. 2020). Contemporary veterinary reviews also recommend keeping IAP as low as is compatible with visualization, typically not exceeding 8–10 mmHg in dogs, with lower effective pressures commonly used in cats (Scott et al. 2020).

Our findings support these recommendations, highlighting that low-pressure pneumoperitoneum (4 mmHg) allows sufficient intra-abdominal visualization and working space while maintaining physiological stability in spontaneously breathing feline patients.

It should be noted that BSVD instruments were reprocessed using ethylene-oxide sterilization. Although EtO_2_ is effective, studies warn of the risk of residual contamination or inadequate reprocessing, especially in complex or single-use instruments, which can compromise sterility despite validated cycles (Josephs-Spaulding and Singh 2020; Owusu et al. 2022). In these circumstances, the use of post-operative antimicrobial coverage—though not standard for clean procedures—was deemed necessary to mitigate potential infection risks.

### Pain

Regarding post-operative pain, scores were higher in the Suture and BVSD groups than in the LOVE group at 1 h post-extubation. At this time point, pain scores in the LOVE group were significantly lower than those recorded in both the Suture and BVSD groups (Dunn’s post-hoc, *p* < 0.05). Nonetheless, 78% of cats in the open surgery groups required rescue analgesia, compared to only one cat in the LOVE group, supporting a clinically meaningful advantage of laparoscopy in the immediate post-operative period. No significant differences were detected at later assessments, most likely due to the confounding effect of rescue analgesia administered immediately after T1 in most Suture and BVSD cases. It is worth noting that the study’s power calculation was based on the primary endpoint of surgical time rather than pain scores; therefore, the sample size may have been underpowered to detect smaller intergroup differences in pain intensity.

Although statistical models including rescue analgesia as a covariate (e.g., mixed-effects or ANCOVA models) could theoretically account for this effect, the limited sample size (*n* = 9 per group) precluded robust multivariate analysis. For this reason, a nonparametric approach (Kruskal–Wallis with Dunn’s post-hoc test and Wilcoxon for within-group comparisons) was adopted to maintain statistical validity, and interpretation beyond the first postoperative hour was restricted to descriptive trends.

Nonetheless, the consistent trend toward lower pain scores and the markedly reduced need for rescue analgesia in the laparoscopic group support the conclusion that the LOVE technique provides superior early postoperative comfort compared with open approaches.

These findings indicate that laparoscopic ovariectomy reduced pain intensity and the need for additional analgesia during the immediate recovery period.

While this difference was most evident within the first 4 h after extubation, further studies with extended monitoring are warranted to confirm whether this advantage persists over a longer timeframe. Other studies previously published concerning the canine species demonstrate that subjects undergoing laparoscopic ovariectomy exhibited lower levels of post-operative pain compared to those undergoing open ovariectomy. Specifically, in a study conducted by Fuertes-Recuero et al. (Fuertes-Recuero et al. 2024), during the first six hours following surgery, dogs that underwent laparoscopic ovariectomy exhibited significantly reduced pain scores in comparison to those subjected undergoing open ovariectomy. In the study by Charlesworth & Sanchez (CHARLESWORTH and SANCHEZ 2019), which involved a comparison of the rates of post-operative complications between dogs undergoing laparoscopic and open ovariectomy, among the complications evaluated, 3% of total complication were attributed to the pain in the open group, whereas no pain-related complications were reported in the laparoscopic group. The results of these studies could be considered to overlap with those obtained in our study on feline species.

The review by Phypers B. (Phypers 2017), despite certain limitations in the available studies considered (e.g. sample size, variability in analgesic protocols), concluded that laparoscopic ovariectomy is associated with more favorable post-operative outcomes, including reduced pain and a lesser impact on activity levels in both dogs and cats.

Gauther et al. (2015), demonstrated that cats undergoing laparoscopic ovariectomy experienced significantly less post-operative pain compared to those treated with median celiotomy or flank laparotomy, and none of the laparoscopic cases exhibited signs of severe pain (Gauther et al. 2015). While these findings established the advantage of laparoscopy over traditional open approaches, the present study extends this knowledge by providing the first prospective randomized comparison of three techniques—open surgery with suture ligation, open surgery with a bipolar vessel-sealing device, and laparoscopic ovariectomy. In addition, our study specifically evaluated the role of BVSD in feline ovariectomy, a modality not previously investigated in this species, and employed a validated pain scoring system with predefined rescue analgesia criteria, thereby strengthening the objectivity and clinical applicability of the results. We can assume that the causes of this reduction in pain found in the laparoscopic approach are the less invasive nature of the technique, both due to the smaller size of the surgical breach, and the less traction applied during the surgical procedures to expose the ovary compared to open techniques.

Moreover, we hypothesize that the use of BVSD, and particularly LOVE, minimized tissue trauma and ovarian pedicle traction, thereby markedly reducing the need for rescue analgesia. In fact, in the present study, only one cat in the LOVE group required rescue analgesia, while only two cats in the BVSD and Suture groups did not require additional analgesia.

Evaluating pain in feline patients within a clinical setting can be challenging, as cats often perceive the veterinary environment as threatening due to its unfamiliar nature and the presence of multiple stress-inducing stimuli (STEAGALL and MONTEIRO 2019). Therefore, immediately after surgery, the cats were placed in a cage in a quiet, silent and dark environment, to eliminate any stressors that could affect the pain assessment. Significant advancements have been made in recent years regarding both the recognition and management of pain in cats. The implementation of validated pain assessment tools has contributed to reducing subjectivity and minimizing observer-related bias during clinical evaluations (Steagall et al. 2022). The most commonly employed scales for assessing acute pain in cats are the Unesp-Botucatu Feline Pain Scale (UFEPS) (Brondani et al. 2011), the Glasgow Feline Composite Measure Pain Scale (CMPS-Feline) (Reid et al. 2017), and the Feline Grimace Scale (FGS) (EVANGELISTA et al. 2019). Each of these scoring systems presents specific strengths and limitations.

For this study the CMPS-Feline scale was employed; this scale demonstrates a high degree of sensitivity, ensuring that cats experiencing pain are accurately identified as needing analgesic intervention (Reid et al. 2017).

Importantly, despite the reduction in surgical time and intraoperative bleeding, the use of a BVSD in open surgery did not result in a measurable reduction in post-operative pain when compared with suture ligation. Both open groups showed similar CMPS-F scores and a high frequency of rescue analgesia, indicating that the main analgesic benefit observed in this study was associated with the laparoscopic approach rather than with the haemostatic technique used in open surgery.

This study presents several limitations that should be considered when interpreting the results. First, 5-mm laparoscopic instruments were employed instead of 3-mm devices. Although 3-mm ports theoretically reduce invasiveness, they are not routinely used in veterinary practice, and vessel-sealing devices of this caliber are not currently available. Moreover, evidence from human laparoscopy indicates that clinically relevant differences in post-operative pain emerge when comparing 5-mm and 10–11-mm instruments, while no substantial differences have been reported between 3-mm and 5-mm devices (ACTON et al. 2016; DONMEZ et al. 2025; GIAMPAOLINO et al. 2024).

Second, insufflation was maintained at 4 mmHg, which is lower than the 8–10 mmHg commonly used in canine and human laparoscopy. This choice was deliberate to minimize cardiorespiratory compromise in cats, whose small abdominal volume and compliant abdominal wall allow adequate visualization at lower pressures (Dorn et al. 2020; Mayhew et al. 2013, Scott et al. 2020; VAN NIMWEGEN and KIRPENSTEIJN 2007).

Third, although strict aseptic technique was applied, bipolar vessel-sealing devices were reprocessed with ethylene oxide sterilization. Literature indicates that reprocessing of complex or single-use instruments may not guarantee complete decontamination, with potential residual contamination or biofilm formation (Josephs-Spaulding and Singh 2020; Owusu et al. 2022). For this reason, post-operative antimicrobials were administered, representing a deviation from standard practice in clean procedures.

Fourth, post-operative pain was assessed only for 4 h after extubation, reflecting the outpatient nature of the procedures. Although this allowed standardized early monitoring, it precluded evaluation of potential differences beyond the immediate recovery period, when CO₂-related peritoneal pain may occur.

Five, It is important to emphasize that operator experience differed among groups, as the open procedures were performed by a surgeon in training under the supervision of a senior clinician, whereas laparoscopic procedures were led by a senior faculty member with extensive experience in minimally invasive surgery. However, to minimize variability, all procedures were conducted under direct supervision, and the same trained assistant participated in all surgeries. Moreover, in the laparoscopic group, only the most critical maneuvers—such as trocar placement and initial abdominal access—were performed exclusively by the experienced surgeon, while the remaining steps followed a standardized protocol across all groups. This structure ensured procedural safety and maintained a comparable level of technical consistency, despite differences in individual operator experience.

An additional limitation of the present study lies in the sample size calculation, which was based on surgical time. While this parameter provided an objective measure of intraoperative performance, it may have resulted in insufficient statistical power to detect differences in postoperative pain among groups. Indeed, although a trend toward lower pain scores was observed in the LOVE group, the study may have been underpowered for this comparison. Considering that postoperative pain is a key welfare indicator and an essential endpoint in the evaluation of surgical techniques, future studies should perform power analyses specifically tailored to this variable, ensuring adequate sample sizes to confirm the observed trends and strengthen the clinical significance of the findings.

Finally, although the pain scales used in the present study have been validated in small animals, there are inherent limitations regarding the interpretation of animal behaviors as painful, which requires a learning curve on the part of the evaluator (CORLETTO 2017).

## Conclusions

Within the limitations of this prospective randomized study, open ovariectomy with suture ligation was associated with longer surgical times, a higher frequency of mild intraoperative bleeding, and greater post-operative analgesia compared to the other techniques. The use of a BVSD in open surgery reduced surgical time and intraoperative bleeding but did not substantially decrease the need for post-operative analgesia. LOVE resulted in shorter incision length, lower pain scores in the immediate post-operative period, and a reduced requirement for rescue analgesia, without prolonging surgical time. These findings support the potential benefits of laparoscopy in improving short-term comfort and perioperative efficiency in feline ovariectomy. However, larger-scale investigations with extended follow-up are needed to confirm the reproducibility and long-term clinical relevance of these results.

## Data Availability

The datasets generated during and/or analysed during the current study are available from the corresponding author on reasonable request.
